# The effect of second-person self-talk on performance and motivation in Japanese individuals

**DOI:** 10.1371/journal.pone.0305251

**Published:** 2024-06-13

**Authors:** Yugo Magata, Ayumi Tanaka

**Affiliations:** Faculty of Psychology, Doshisha University, Kyoto, Japan; Kitami Institute of Technology, JAPAN

## Abstract

Talking to oneself using the second-person pronoun [“you” has been shown to enhance performance and autonomous motivation in English speakers. However, there is a lack of evidence on whether it can be replicated for speakers of other languages, such as Japanese, in which the grammatical subject is usually omitted in daily conversation. Based on self-determination theory, the present study examined the effects of second-person self-talk for a sample of Japanese individuals on task performance, intrinsic motivation, and three styles of extrinsically motivated regulations: identified, introjected, and external. We randomly assigned 411 undergraduate students to either an experimental group (second-person self-talk, first-person self-talk, and non-subject self-talk) or a control group. An anagram task was used to assess performance. No significant difference was found between the four groups in intrinsic motivation or performance. For extrinsic regulations, the results showed that first-person self-talk led to higher external regulation than non-subject self-talk and the control group. The possible reason for contradictory findings with our hypothesis and implications have been discussed.

## Introduction

“I don’t want to go to school,” “I hate this work, and I don’t know what I want to do”—there are motivational problems in society that lead to performance issues such as dropout and low productivity. To enhance motivation, parents or bosses can provide a meaningful rationale, acknowledge the person’s feelings, or provide choices, which have been referred to as autonomy support according to self-determination theory [1]. However, people do not always have a nurturing parent, teacher, or boss who can provide such support. Studies [2, 3] have found that motivation can be enhanced by self-talk—talking to oneself. Surprisingly, certain patterns of language use in self-talk have been shown to impact motivation. Specifically, self-talk using the second-person pronoun “you,” such as “You can make it!” instead of the first-person pronoun “I,” enhances performance [2, 4–6] and motivation [2]. However, most studies on self-talk using “you” have been conducted with English speakers, and sufficient evidence to support comparable effects in speakers of other languages is lacking. This study addresses this gap in the existing literature. The present study examines the effect of self-talk with second-person pronouns (called “second-person self-talk”) on performance, intrinsic motivation, and different types of extrinsic motivation.

*Intrinsic motivation* refers to actions for the inherent satisfaction of the action and is, therefore, fully autonomous; on the other hand, extrinsic motivation refers to actions for gaining outcomes [7]. Intrinsic motivation, or wholly autonomous motivation [1], is the key to solving several motivational problems [1]. One study [2] showed that participants who used second-person self-talk were intrinsically motivated for anagram tasks and solved more anagrams compared to those who used self-talk with the first-person pronoun (hereinafter “first-person self-talk”). Beyond its influence on intrinsic motivation, self-talk using non-first-person pronouns and one’s own name (called “non-first-person self-talk”) promotes successful emotion regulation and self-control compared with first-person self-talk. For example, participants who engage in self-talk with non-first-person pronouns feel less negative emotion when remembering negative events [8], think capable of coping with the demands of a stressful environment [4], have reduced emotional reactivity to the aversive images without cognitive control [9], and choose the healthier option between healthy and unhealthy food [5].

Dolcos and Albarracin [2] discussed that the use of second-person self-talk could facilitate self-regulation because it is derived from successful social regulation. That is, the language used in self-talk could be modeled after supportive sentences from parents and teachers. The socio-genetic perspective suggests that initial external instructions and encouragements (e.g., you must stay focused) associated with successful behavior regulation could have been personalized and internalized into the individual [10]. It may be applied similarly in the situations that require self-regulation [2].

Research on self-talk within the framework of self-determination theory is scarce [11–13]. One recent study recognized the effect of second-person self-talk [11], and they focused on the delivery of the self-talk. The effects of the second-person self-talk in autonomy supportive style and self-controlling style were compared; the former was found to be related to positive emotion. A similar comparison was made in other studies [12, 13]. However, more research would be needed on the association between the difference in pronoun use in self-talk and autonomous motivation.

Studies concerning second-person self-talk have mostly been conducted with English speakers who do not abbreviate grammatical subjects. However, in some languages, such as Japanese, the grammatical subject is often omitted in conversation. For example, Japanese people usually say “できるよ”([You] can do it) instead of “あなたはできるよ.” (You can do it). The sentences are identical in meaning, although only the second includes the pronoun “you” explicitly. The languages that allow the omission of pronouns are more likely used in cultures that consist of uncertainty avoidance rather than risk tolerance, determining a person’s worth based on one’s own background characteristics such as family rather than on personal achievement, and collectivistically rather than individualistically [14]. It has consistently been found that Japanese people view “self” as interdependent with others rather than independent from society [15]. It should be noted, however, that independence and autonomy is a different concept [1, 16]. Most studies showed that autonomous motivation predicts goal attainment in any type of culture, including Japan [17, 18].

One empirical study by Simizu et al. [6] has examined the difference in the effect of self-talk among the Japanese by comparing the effect of first-person and non-first-person self-talk on a self-control task. They found that participants who used non-first-person pronouns showed longer persistency in holding the handgrip compared to those who used first-person pronouns in the group in which the task focused on positive outcomes. However, no difference was found when the focus was on negative outcomes. Their results imply that the change in pronoun usage has certain impacts in the Japanese context. However, the main effect of pronoun usage is not evident since their experimental design focused on the moderation effect of regulatory focus. In fact, the main effect of self-talk in the study was not significant, and the moderation effect was rather small. Moreover, the effect on intrinsic motivation was not clear. Therefore, it is worth evaluating whether the results obtained by Dolcos and Albarracin [2] can be replicated in a Japanese sample.

Most prior studies on the effect of self-talk with second-person pronouns have compared the effect with that of first-person self-talk [4–6]. In our study, we added the group in which the self-talk omitted the grammatical subject of the sentence (called “non-subject self-talk”). A control group was also added to examine the effectiveness of self-talk. We hypothesized that second-person self-talk enhances task performance and intrinsic motivation compared to first-person self-talk, non-subject self-talk, and the control group.

Further, we examined whether second-person self-talk influences three types of extrinsically motivated regulation defined in the self-determination theory: identified, introjected, and external regulations [7]. They differ in the level of autonomy for the action and yet can coexist within a single activity [1]. Identified regulation concerns the action in which a person consciously identifies the value and has a relatively high degree of autonomy [1]. Introjected regulation concerns the action driven by internal rewards of self-esteem for success and by avoidance of anxiety, shame, or guilt. The value of action based on introjected regulation is partially internalized; therefore, introjected regulation is not fully controlled but is somewhat controlled by oneself. External regulation concerns the action driven by the anticipation of extrinsic reward or punishment and is non-autonomous. Second-person self-talk should positively influence relatively autonomous and adversely influence relatively controlled motivation regulations. The present study explores the effect of groups on identified, introjected, and external regulations. To our knowledge, no previous research has examined the effect. The self-determination theory perspective would help to further understand the mechanism of the effect of pronoun difference in self-talk.

## Materials and method

This study was preregistered at the OSF (https://osf.io/bupef), and data is available at https://osf.io/3b85t/.

### Participants

We aimed for a sample size of 74 participants per group (296 total) based on a power analysis for ANCOVA in G*Power 3.1.9.7 [19] using an alpha of .05, power of .80, and effect size η^2^ = 0.04 obtained from our pilot study. A total of 1136 individuals were recruited for the study. Among the initial pool of participants, 37 were firstly recruited from a psychology class at a university, and 1099 were secondly recruited by a research corporation to obtain the targeted sample size. Of the 1099 participants, 10 were excluded for either failing to report their mother tongue or for indicating it to be non-Japanese. Participants from the psychology class were provided with a prepaid card worth 500 yen (3.50–4.50 USD) as compensation for their participation, while those from the research corporation received compensation based on their contractual agreement for their involvement in the study. After applying the exclusion criteria as described later and explained in S1 Table, 411 participants (261 men, 147 women) were included in the analyses. All were native Japanese speakers, and their ages ranged from 18 to 30 years (M = 20.51, standard deviation, SD = 1.26). Of those participants, 382 were from the company and 29 from the university.

### Anagram task

**Fig 1:**
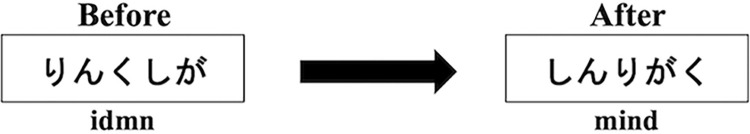
Example of an anagram task.

The anagram task was used twice (baseline and main) as an experimental task in this study. The task was to rearrange five characters from the hiragana syllabary, which represent single vowels or the combination of a consonant and a vowel in each meaningless string to form a word (Fig 1). Five strings were presented simultaneously on the screen. The anagram tasks were adopted from a database of five-character hiragana anagrams [20]. In the baseline section, participants solved five anagram tasks at once in five minutes; in the main section, they solved 10 anagram tasks at once in ten minutes.

### Task for controlling language ability

We used a remote association task (RAT) [21] to control language ability. This task requires finding a common Kanji character that is combined with each of the three Kanji presented as question words to form a word. The RAT was presented one at a time. Participants solved one RAT for precisely one minute and three RATs in total. The RAT was adopted from the database of a Japanese version of RATs [22].

### Measurement of motivation for the anagram task

Motivation was measured twice in this study: at the baseline and main sections using the Self-Regulation Questionnaire for Academic Activity [18]. This scale is based on self-determination theory and has four sub-scales: intrinsic, identified, introjected, and external regulations. We mainly focused on intrinsic regulation. The other three regulations were measured in the exploratory analysis. The scale originally measured the academic motivation of high school students [18] and also applied for college students [23]. The scale consists of 20 items; we chose 13 to shorten participation time. To use the scale in the current context, we modified the instructions and the items (Table 1). The participants were asked, “Please indicate to what extent each of the following items corresponds to one of the reasons for which you were solving the task.” They responded, for example, to “Because I wanted to do the task,” which was modified from the original item, “Because I want to study.” Responses were obtained on a four-point Likert-type scale (1 = “not at any time” to 4 = “always”). We described intrinsic regulation in the baseline section as “intrinsic regulation (baseline)” and in the main section as “intrinsic regulation.” Additionally, we asked participants whether monetary reward was the reason for their participation. This item was included as an external regulation. The sub-scale of external regulation shows low reliability (α = .55). However, it consists of four items, and α > .50 has sometimes been assumed to be moderate reliability [e.g., 24, 25]. We retained the external regulation sub-scale for further analysis, considering the importance of examining this quite common form of motivation [1]. More detailed psychometric information of the measure can be found in S1 Fig, S2 Table.

**Table 1:** Items for the motivation measure.

Intrinsic regulation (α = .85)	Because I enjoyed solving the task
	Because I enjoyed taking up a difficult challenge.
	Because it is enjoyable to discover new ways of solving the task.
	Because I wanted to do the task.
Identified regulation (α = .79)	Because this experience leads to success in the future.
	Because working on the task was for my own good.
	Because working on the task was important.
Introjected regulation (α = .80)	Because I did not want to lose other participants on the task.
	Because I want to get higher scores than other participants do.
	Because I feel miserable if I can’t perform better.
External regulation (α = .55)	Because people around me asked me to do so.
	Because solving the problem was something like a rule.
	Because everyone else solved the task as if that was just what they were supposed to do.
	Because I knew that I could get a reward for solving the task.

The items are modified from the Self-Regulation Questionnaire for Academic Activity. English version of the Self-Regulation Questionnaire for Academic Activity was obtained from the author, T. Nishimura (personal communication, November 9, 2023).

### Measurement of tiredness and experience with the task

We assessed the level of tiredness since performance is likely to be impaired after a cognitive load task [26]. This potential impairment could influence the subsequent performance and motivation in the main section’s anagram task. Participants were asked to rate their tiredness level on a 4-point Likert-type scale, ranging from 1 (“not feeling at all”) to 4 (“feeling strongly”). Additionally, they were asked to rate their experience with the anagram task on an 11-point Likert-type scale ranging from 1 (“not at all”) to 11 (“always”).

### Procedure

This survey was conducted between January 20 and 30, 2022, and between March 15 and 24, 2022. The experimental procedure was approved by the local ethical committee (KH21092, KH21096). The authors had no access to the participants’ personally identifiable information during or after data collection.

**Fig 2:**
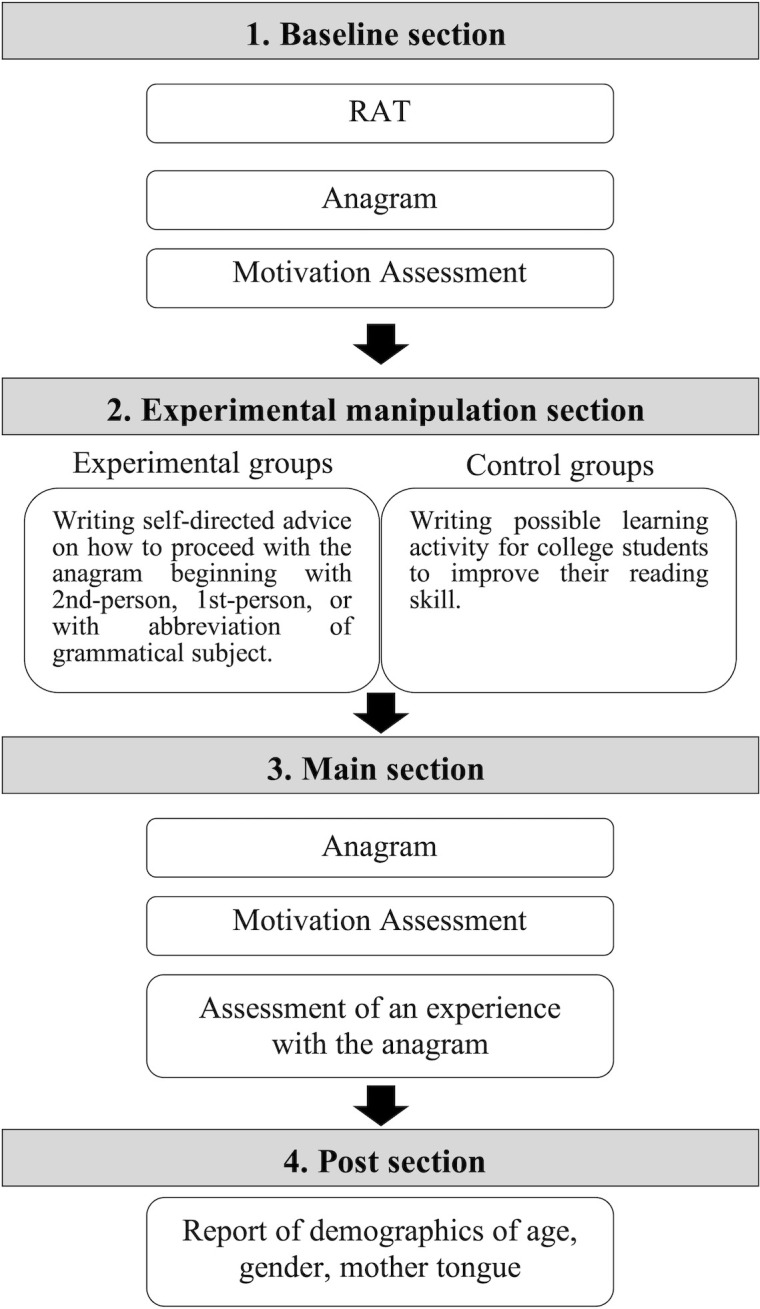
Flow of the procedure.

The procedure consisted of four sections: baseline, experimental manipulation, main, and post (Fig 2). Participants were provided with general instructions about the purpose of the experiment, procedure, and timeline of the study. They were informed that their participation was voluntary and that they could withdraw their consent at any moment. Subsequently, they provided their consent to these terms.

First, in the baseline section, participants sequentially completed the RAT, followed by the anagram task, and then responded to the items measuring their motivation behind participating in the anagram task. Then, they were given time to review the answers to the anagram task.

Second, in the experimental manipulation, participants were randomly assigned to one of the four groups: three experimental groups (second-person, first-person, and non-subject self-talk) and one control group using Randomizer in Qualtrics. The experimental groups were instructed to prepare for the following anagram task by writing self-directed advice on how to proceed with the task. The second-person self-talk group was asked to write an advisory note beginning with “You,” the first-person self-talk group with “I,” and the non-subject self-talk group with no subject of the sentence. The control group was asked to write a possible learning activity for college students to improve their reading skills. Three examples of advisory notes were presented to the experimental groups, and three examples of possible learning activities for college students were presented to the control group. An example of advice is, “You might want to move the first letter of a string to the end” (in the second-person group). One hundred and twenty-four participants were excluded from the statistical analyses as they did not follow the experimental instructions (See S1 Table). One advisory note or one possible learning activity was required to be written in one sentence, and the range of the number of notes or possible learning activities was 1–8. The exact instructions are described in the S3–S5 Tables. We measured the number and time of advisory notes or possible learning activities. Five hundred twelve participants who disengaged before writing the self-talk or learning activity were excluded from statistical analysis. Participants were asked to indicate the extent of their tiredness after engaging in self-talk.

Third, in the main section, participants were asked to solve the anagram task again. Forty-one participants who disengaged before completing the anagram task were excluded from the statistical analysis, resulting in 572 participants. Then, participants answered the same motivation measuring scale for the anagram task. Additionally, they rated their experience with the anagram task.

Finally, in the post-task section, participants were asked to report their demographics: age, gender, mother tongue, and the possible objective of this study. No participant realized the objective of this study. We measured the time participants took to complete the procedures. Those who took an excessively long time were excluded (see S1 Table), and 411 participants were included for statistical analysis.

### Statistical analyses

We set the significant level as p = .05 for all statistical analyses. There was no statistically significant difference in intrinsic regulation, t(409) = 1.08, p = .28, and in anagram performance, t(409) = 0.79, p = .43 between participants from the research company and those from the university. Therefore, the two sets of data were analyzed together.

For analysis, we used R 4.2.1 [27] and followed our preregistered plan. We reported a descriptive analysis of all variables and used analysis of covariance (ANCOVA) to examine our hypothesis, with intrinsic regulation and anagram performance as dependent variables and group as the independent variable (second-person vs. first-person vs. non-subject vs. control). We used Tukey’s honestly significant difference (HSD) test for multiple comparisons. Covariates were RAT, anagram performance (baseline) or intrinsic regulation (baseline), and tiredness. For further information on the reason for the unused variables as covariates, refer to the S1 Text.

Additionally, we conducted an exploratory analysis to examine the effect of the group on identified, introjected, and external regulations using ANCOVA. Covariates were RAT, each regulation (baseline), and tiredness.

## Results

### Descriptive statistics

On average, participants took 175 seconds (SD = 189.97) for self-talk, wrote 4.43 sentences (SD = 2.52), and were not very experienced with anagrams (M = 2.20, SD = 1.80). The descriptive statistics and Pearson’s correlation between variables that were used to test our hypothesis are shown in Table 2, and correlations between all variables are shown in Table A in S1 Text. Performance in RAT had a weak but statistically significant correlation with that in the anagram task, r = .16, p = .00, but not with intrinsic regulation (r = .02, p = .73). Tiredness showed a significant correlation with intrinsic regulation, r = -.22, p = .00, but not with anagram performance, r = -.07, p = .17.

**Table 2:** Descriptive statistics and correlations for variables.

	M	SD	1	2	3	4	5	6	7	8	9	10	11
Baseline section													
1. Intrinsic regulation	2.75	0.69											
2. Identified regulation	1.88	0.66	.37*										
3. Introjected regulation	2.03	0.77	.32*	.48*									
4. Extrinsic regulation	2.17	0.58	- .07	.32*	.40*								
5. Anagram performance	2.70	1.37	.26*	- .04	.06	- .06							
Main section													
6. Intrinsic regulation	2.59	0.78	.72*	.31*	.27*	- .04	.17*						
7. Identified regulation	1.77	0.71	.29*	.77*	.45*	.36*	-. 15*	.39*					
8. Introjected regulation	1.94	0.80	.27*	.41*	.81*	.39*	.02	.37*	.53*				
9. Extrinsic regulation	2.15	0.62	- .02	.29*	.38*	.75*	- .07	.09	.45*	.51*			
10. Anagram performance	5.54	2.73	.13*	- .11*	.02	- .14*	.41*	.33*	- .06	.06	- .07		
Control variables													
11. RAT	1.28	0.91	- .03	- .10*	- .02	- .06	.13*	.02	- .07	- .02	- .01	.16*	
12. Tiredness	2.75	0.75	- .20*	- .04	- .13*	.08	- .03	- .22*	- .02	- .08	.02	- .07	- .06

*p < .05. RAT, remote association task; SD, standard deviation.

### Group difference at baseline

There was a statistically significant difference between groups in the RAT (F_(3,407)_ = 2.68, p = .047). However, by multiple comparisons, no significant difference was found between any two groups. At baseline, there was no significant difference between groups in anagram performance (F_(3,407)_ = 0.16, p = .92). However, there was a significant difference in intrinsic regulation (F_(3,407)_ = 3.11, p = .03). Furthermore, there was no significant difference between groups in tiredness (F_(3,407)_ = 1.61, p = .19). Multiple comparisons revealed that the first-person self-talk group had significantly higher intrinsic regulation (baseline) than the control group (p = .04).

### The effect of self-talk on anagram performance and intrinsic regulation

**Fig 3:**
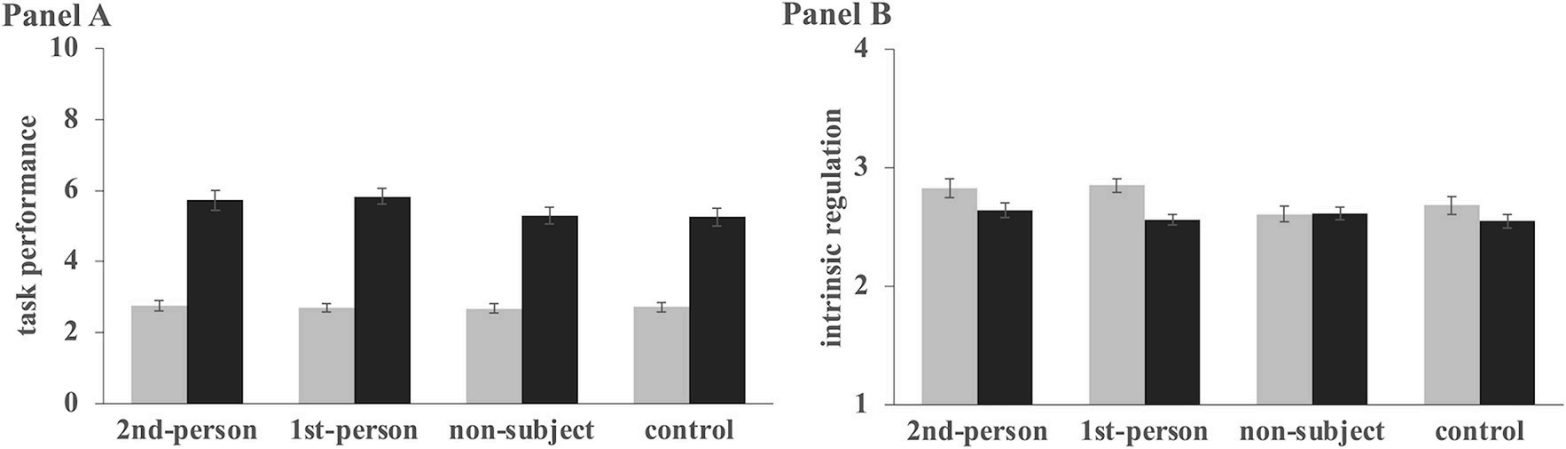
The effect of self-talk on anagram performance and intrinsic regulation. Error bars show 95% confidence intervals (CIs).Anagram performance (Panel A) and intrinsic regulation (Panel B) scores of each group are shown for the baseline and main sections. The maximum points were five and 10 at baseline and main sections, respectively, in the anagram task.

The ANCOVA to examine the effect of self-talk on anagram performance and intrinsic regulation revealed no significant group effect in both anagram performance, F(3,404) = 1.54, p = .21, and intrinsic regulation, F(3,402) = 0.64, p = .59 (Fig 3).

### Exploratory analysis: The effect of self-talk on identified, introjected, and external regulations

**Fig 4:**
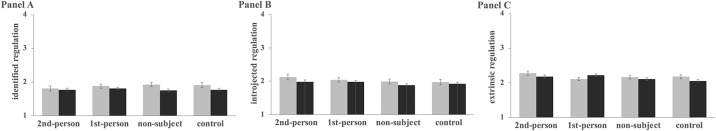
The effect of self-talk on extrinsic motivation. Error bars show 95% confidence intervals (CIs). Identified regulation (Panel A), introjected regulation (Panel B), and external regulation (Panel C) scores for each group are shown for baseline and main sections.

The ANCOVA showed no statistically significant differences between groups in identified regulation, F(3,400) = .33, p = .80, and introjected regulation F(3,400) = 1.21, p = .31. However, it showed group effect on external regulation F(3,402) = 3.58, p = .01(Fig 4). Multiple comparisons showed that the first-person self-talk group had significantly higher external regulation than the control group p = .01.

## Discussion

The present study compared performance and the intrinsic motivation for an upcoming task after second-person self-talk with that after first-person self-talk, non-subject self-talk, and in control groups of Japanese individuals. We hypothesized that second-person self-talk enhances task performance and intrinsic motivation compared to first-person self-talk, non-subject self-talk, and the control group.

The findings showed that second-person self-talk did not have any significant effect on task performance and intrinsic motivation compared to the other three groups. There are two possible interpretations of this result. First, second-person self-talk may not influence intrinsic motivation and task performance in Japanese individuals. Motivational problems are a matter of concern in society [28–30], and using second-person self-talk could have been a prospective solution to this problem [2]. However, the results did not support this possibility for Japanese individuals. It could be a problem with the experimental paradigm. Therefore, we may need to examine the effect of second-person self-talk on task performance and intrinsic motivation using the present study paradigm for a sample of English-speaking individuals.

Second, the contents of second-person self-talk may be better in their abstract form than in their concrete form. In this study, participants were instructed to use concrete advice, such as “You should move the first letter of the string to the end,” rather than abstract advice, such as “You should try hard.” According to Trope et al. [31], relatively abstract, coherent, and superordinate mental representations of action or concept create psychological distance from the self. Researchers have explained the effect of talking to oneself using the second-person pronoun “you” is caused by the psychological distance from the self [5]. Therefore, second-person self-talk could increase accessibility to abstract mental representations, which suggests it may be better suited to abstract nature. Future studies should examine whether the difference in the level of abstractness of self-talk content modulates the influence.

Exploratory analysis showed that second-person self-talk did not influence any of the three extrinsic motivations defined in the self-determination theory (external, introjected, and identified regulations). It is worth noting that the first-person self-talk group was more engaged in the task through external regulation and had the least autonomous motivation compared to the control group. The result may be related to that of prior studies that showed excessive use of first-person pronouns was associated with negative factors such as depression [32, 33]. Excessive use of first-person pronouns could have negative consequences on motivation and well-being. To our knowledge, this exploratory analysis is the first to show that pronoun differences used in self-talk influenced motivation based on the self-determination theory perspective. However, the results should be interpreted with caution as the effect size was small.

Several limitations to our study need to be considered when interpreting the results. First, the use of personal pronouns as a grammatical subject seemed unnatural for Japanese participants. In this study, writing self-directed advice about the way of proceeding with subsequent tasks was operationally interpreted as self-talk. Eighty-five participants in the second- and first-person groups failed to use the assigned personal pronoun in self-talk even though they answered the content-checking questions correctly, while no participants failed to follow the instructions in the non-subject group. Japanese people may have difficulty engaging in self-talk with the grammatical subjects. Second, reported self-talk strategies varied across participants. The differences in the content of the strategies may be confounded with the results. The difference in the effectiveness of the strategy for doing the task might have a stronger influence than the difference in the pronoun. Third, seven items were removed as three supplementary but important points, and wordings were modified from the original motivation scale. The removal may have reduced the scale variance, and wording modifications may have led to some participants misinterpreting that they were not being asked about their motivation for engaging in the task but for participating in the study. These issues may have been related to the lack of significant results. Also, even though randomization was conducted and the baseline was controlled for hypothesis testing, group differences in intrinsic motivation at baseline might have been problematic.

In conclusion, this was the first study to investigate whether second-person self-talk, which has been effective for English speakers, also influences the task performance and motivation of Japanese speakers who often omit the subject in everyday conversation. The findings did not support the hypothesis. Future studies should use the same experiment to verify whether the present findings are replicated in an English-speaking sample. In future studies, the moderating influence of self-talk according to the level of content abstractness should be examined.

## Supporting information

S1 Table Specific number of participants and rationale for exclusion by each section. (DOCX)

S2 Table Psychometric information of the motivation measure. (DOCX)

S3 Table Instructions for second- and first-person self-talk groups in the experimental manipulation. (DOCX)

S4 Table Instructions for the non-subject self-talk group in the experimental manipulation. (DOCX)

S5 Table Instructions for the control group in the experimental manipulation. (DOCX)

S1 Fig Histogram of motivation measure. (TIF)

S1 Text Correlations between variables and reasons for unused variables in data analysis. (DOCX)
