# Supplementary material for: The effect of second-person self-talk on performance and motivation in Japanese individuals
Source: PLoS One. 2024 Jun 13;19(6):e0305251. doi: 10.1371/journal.pone.0305251 (PMC11175409; doi:10.1371/journal.pone.0305251)
Supplement: S1 Table — (DOCX) [file pone.0305251.s001.docx]

**S1 Table. Specific number of participants and rationale for exclusion by each section.**

| Status of participants | Rationale for exclusion | Excluded number |
| --- | --- | --- |
| Did not qualify for participating in the research | Did not report or indicated a language other than Japanese as the mother tongue | 10 |
| Did not complete the experimental procedure | Disengagement before writing a self-talk or learning activity | 512 |
|  | Disengagement before anagram task | 46 |
| Completed the experiment | Writing an advice about daily life (e.g., “I had better sleep early.”, “I better live my life more seriously.”) | 39^a^ |
|  | Incorrect pronoun usage in advice (e.g., using “I” under the condition instructed to use “You”.) | 85^b^ |
|  | Taking longer time to complete the tasks (i.e., outliers) | 37^c, d^ |

^a^ Number of excluded participants for writing an advice about daily life instead of anagram task were not statistically different between groups ($\chi^{2}$(2) = 4.42, p = .11, 2nd-person = 7, 1st-person = 15, non-subject = 17).

^b^ Number of excluded participants for not using the correct pronoun were not statistically different between groups ($\chi^{2}$(1) = 1.01, p = .31, 2nd-person = 45, 1st-person = 40).

^c^ In pre-registration, the time limit was 3600 seconds. However, we changed the criterion to 9822 seconds using the criterion of adjusted boxplot for distribution [Huuberta M, Vandervieren E. An adjusted boxplot for skewed distributions. Comput Stat Data Anal. 2008;52: 5186–5201.] using “robustbase” R package. This is because 12.28% of participants would be excluded under the 3600 seconds criterion, and this exclusion rate is considered too high. The exclusion was not statistically different in four groups ($\chi^{2}$(3) = 2.13, p = 0.55, 2nd-person = 10, 1st-person = 13, non-subject = 9, control = 5).

^d^ Among the participants who completed the research, remote association task (RAT), anagram performance (baseline), and intrinsic regulation (baseline) was compared between participants included in the data analysis and those excluded from the data analysis. Participants who were not excluded from the data analysis scored significantly higher on the RAT, t(570) = 3.22, p = .00, anagram performance (baseline), t(570) = 5.43, p = .00, and intrinsic regulation (baseline), t(568) = 4.77, p = .00, compared to those who were excluded.
