# Supplementary material for: The effect of second-person self-talk on performance and motivation in Japanese individuals
Source: PLoS One. 2024 Jun 13;19(6):e0305251. doi: 10.1371/journal.pone.0305251 (PMC11175409; doi:10.1371/journal.pone.0305251)
Supplement: S2 Table — (DOCX) [file pone.0305251.s002.docx]

| **S2 Table. Psychometric information of the motivation measure.** | | | | | | | | |
| --- | --- | --- | --- | --- | --- | --- | --- | --- |
|  |  | *M* | *SD* | | *Skewness* | *Kurtosis* | *Floor effect* | *Ceiling effect* |
| Intrinsic regulation | Because I enjoyed solving the task | 2.86 | 0.83 | | - 0.45 | - 0.26 | 0.07 | 0.21 |
|  | Because I enjoyed taking up a difficult challenge. | 2.78 | 0.80 | | - 0.42 | - 0.16 | 0.07 | 0.16 |
|  | Because it is enjoyable to discover new ways of solving the task. | 2.62 | 0.83 | | - 0.19 | - 0.50 | 0.09 | 0.13 |
|  | Because I wanted to do the task. | 2.73 | 0.86 | | - 0.28 | - 0.56 | 0.09 | 0.18 |
| Identified regulation | Because this experience leads to success in the future. | 1.67 | 0.68 | | 0.71 | 0.10 | 0.45 | 0.01 |
|  | Because working on the task was for my own good. | 1.91 | 0.80 | | 0.46 | - 0.53 | 0.34 | 0.03 |
|  | Because working on the task was important. | 2.07 | 0.86 | | 0.42 | - 0.53 | 0.27 | 0.06 |
| Introjected regulation | Because I did not want to lose other participants on the task. | 2.07 | 0.91 | | 0.39 | - 0.78 | 0.31 | 0.07 |
|  | Because I want to get higher scores than other participants do. | 2.11 | 0.92 | | 0.28 | - 0.95 | 0.31 | 0.07 |
|  | Because I feel miserable if I can’t perform better. | 1.91 | 0.90 | | 0.49 | - 0.92 | 0.41 | 0.04 |
| External regulation | Because people around me asked me to do so. | 1.54 | 0.79 | | 1.20 | 0.25 | 0.64 | 0.01 |
|  | Because solving the problem was something like a rule. | 2.12 | 0.92 | | 0.22 | - 1.03 | 0.31 | 0.06 |
|  | Because everyone else solved the task as if that was just what they were supposed to do. | 2.07 | 0.94 | | 0.25 | - 1.16 | 0.36 | 0.05 |
|  | Because I knew that I could get a reward for solving the task. | 2.96 | | 0.91 | - 0.76 | - 0.10 | 0.10 | 0.29 |

The items are modified from the Self-Regulation Questionnaire for Academic Activity. English version of the Self-Regulation Questionnaire for Academic Activity was obtained from the author, T. Nishimura (personal communication, November 9, 2023).

Floor / Ceiling effects are the proportion of respondents scoring lowest (floor) or the highest (ceiling) possible score across a given domain.
