# Supplementary material for: The effect of second-person self-talk on performance and motivation in Japanese individuals
Source: PLoS One. 2024 Jun 13;19(6):e0305251. doi: 10.1371/journal.pone.0305251 (PMC11175409; doi:10.1371/journal.pone.0305251)
Supplement: S4 Table — (DOCX) [file pone.0305251.s004.docx]

**S4 Table. Instructions of non-subject self-talk group in the experimental manipulation.**

| Contents | Instruction |
| --- | --- |
| Introduction and purpose | One of the focuses of this study is on “self-directed advice.” After a while, you will be asked to solve the same puzzle as before (the puzzle that involved rearranging a string of five letters that have no meaning to form a meaningful word). However, before that, please think about some advice for yourself. This is because we are interested in how people prepare themselves for challenging puzzles and how each type of self-preparation affects performance. |
| Prompt for engaging self-talk without grammatical subject | In everyday life, you may give some advice to someone who is engaging in a task. Here, please think about some advice for yourself for working on the puzzle (the puzzle that involved rearranging a string of five letters that have no meaning to form a meaningful word). Moreover, individuals advise themselves by omitting the grammatical subject, so we would like you to do this.  Please try to write advice about the puzzle by omitting grammatical subject as much as possible. |
| Provision of format of self-talk | For example, advise yourself, “should read all puzzles first.” Please refer to the following example of “advice to yourself.”  An example of advice to yourself would be:   - Try to make a noun with three out of five letters. - Try to focus on the letters that are far apart. - Try to think about whether you feel something by the sound. - Try to write the word that comes to your mind. |
| Additional instruction how to write down self-talk | We will ask you to write down advice on the next page. Each “advice to yourself” should be written in one sentence, and you should write no more than eight and no less than one. Take at least 1 min to write the advice. The next page is where you write advice, but the → button will not appear on the assignment response page until 1 min has elapsed. Once you understand the instructions, answer the confirmation questions below and press “→” to proceed to the assignment response page. |
