# Supplementary material for: The effect of second-person self-talk on performance and motivation in Japanese individuals
Source: PLoS One. 2024 Jun 13;19(6):e0305251. doi: 10.1371/journal.pone.0305251 (PMC11175409; doi:10.1371/journal.pone.0305251)
Supplement: S5 Table — (DOCX) [file pone.0305251.s005.docx]

**S5 Table. Instructions for the control group in the experimental manipulation.**

| Contents | Instruction |
| --- | --- |
| Introduction and purpose | One of the focuses of this study is on “reading comprehension.” After a while, you will be asked to write educational activity for improving the reading skills of college students. |
| Prompt for writing educational activity | Consider what kind of education should be provided at the university to enhance college students' reading comprehension skills. However, the educational activity does not have to be verified to be actually effective.  Please try to write an educational activity as much as possible. |
| Provision of format and example of educational activity | Please refer to the following examples of “educational methods to improve reading comprehension of college students.” An example of advice to yourself would be:   - A basic course in linguistics as a required class - In each class, have students read a book and submit an assignment to make a summary of the book. - Practice speed-reading in seminars. |
| Additional instruction how to write down educational activity | We will ask you to write down an educational activity on the next page. Each “educational activity” should be written in one sentence, and you should write no more than eight and no less than one. Take at least 1 min to write the educational activity. The next page is where you write educational activity, but the → button will not appear on the assignment response page until 1 min has elapsed. Once you understand the instructions, answer the confirmation questions below and press “→” to proceed to the assignment response page. |
