# Supplementary figures and images for: The effect of second-person self-talk on performance and motivation in Japanese individuals
Source: PLoS One. 2024 Jun 13;19(6):e0305251. doi: 10.1371/journal.pone.0305251 (PMC11175409; doi:10.1371/journal.pone.0305251)

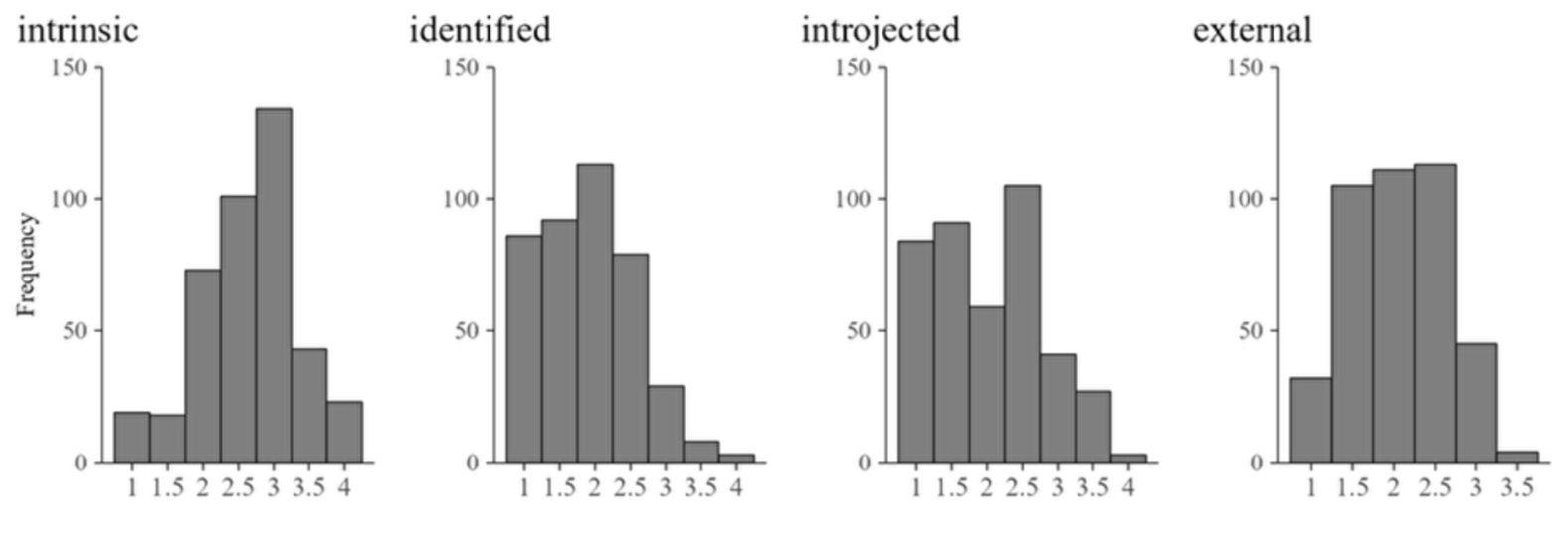

Supplement: S1 Fig — (TIF) [file pone.0305251.s006.tif]
