# Supplementary material for: The effect of second-person self-talk on performance and motivation in Japanese individuals
Source: PLoS One. 2024 Jun 13;19(6):e0305251. doi: 10.1371/journal.pone.0305251 (PMC11175409; doi:10.1371/journal.pone.0305251)
Supplement: S1 Text — (DOCX) [file pone.0305251.s007.docx]

**S1 Text. Correlations between variables and reasons for unused variables in data analysis.**

Table A shows correlations between all variables. Variable “number of sentences” indicates the number of self-talks in the experimental groups or the number of possible learning activities in the control group. Variable “time for writing” indicates the time participants took for writing self-talk or possible learning activity.

As in Table A, the number of sentences was significantly correlated with the anagram performance; however, the correlation coefficient was small (r = -.13, p = .01). The time for writing, total time, the anagram experience, and age did not significantly correlate either with anagram performance or intrinsic regulation. Therefore, these measures were not considered as covariates.

**Table A. Correlations between all variables.**

|  | 1 | 2 | 3 | 4 | 5 | 6 | 7 | 8 | 9 | 10 | 11 | 12 | 13 | 14 | 15 | 16 |
| --- | --- | --- | --- | --- | --- | --- | --- | --- | --- | --- | --- | --- | --- | --- | --- | --- |
| Baseline |  |  |  |  |  |  |  |  |  |  |  |  |  |  |  |  |
| 1. Intrinsic |  |  |  |  |  |  |  |  |  |  |  |  |  |  |  |  |
| 2. Identified | .37* |  |  |  |  |  |  |  |  |  |  |  |  |  |  |  |
| 3. Introjected | .32* | .48* |  |  |  |  |  |  |  |  |  |  |  |  |  |  |
| 4. Extrinsic | - .07 | .32* | .40* |  |  |  |  |  |  |  |  |  |  |  |  |  |
| 5. Anagram performance | .26* | - .04 | .06 | - .06 |  |  |  |  |  |  |  |  |  |  |  |  |
| Main section |  |  |  |  |  |  |  |  |  |  |  |  |  |  |  |  |
| 6. Intrinsic | .72* | .31* | .27* | - .04 | .17* |  |  |  |  |  |  |  |  |  |  |  |
| 7. Identified | .29* | .77* | .45* | .36* | - .15* | .39* |  |  |  |  |  |  |  |  |  |  |
| 8. Introjected | .27* | .41* | .81* | .39* | .02 | .37* | .53* |  |  |  |  |  |  |  |  |  |
| 9. Extrinsic | - .02 | .29* | .38* | .75* | - .07 | .09 | .45* | .51* |  |  |  |  |  |  |  |  |
| 10. Anagram performance | .13* | - .11* | .02 | - .14* | .41* | .33* | - .06 | .06 | - .07 |  |  |  |  |  |  |  |
| Other variables |  |  |  |  |  |  |  |  |  |  |  |  |  |  |  |  |
| 11. RAT ^a^ | - .03 | - .10* | - .02 | - .06 | .13* | .02 | - .07 | - .02 | - .01 | .16* |  |  |  |  |  |  |
| 12. Tiredness | - .20* | - .04 | - .13* | .08 | - .03 | - .22* | - .02 | - .08 | .02 | - .07 | - .06 |  |  |  |  |  |
| 13. Number of sentences ^b^ | .02 | .16* | .04 | .09 | - .05 | - .03 | .16* | - .04 |  | - .13* | .02 | .02 |  |  |  |  |
| 14. Time for writing ^c^ | - .02 | .03 | - .05 | - .02 | - .04 | - .04 | - .01 | - .06 | - .06 | - .08 | - .03 | .04 | .20* |  |  |  |
| 15. Total time | .03 | .00 | .05 | .07 | - .01 | .03 | .01 | .06 | .02 | .03 | .02 | .01 | .09 | .25* |  |  |
| 16. Anagram experience | .00 | .11* | .07 | .10* | .05 | .01 | .13* | .12* | .06 | .01 | - .01 | - .05 | .09 | .07 | .04 |  |
| 17. Age | .02 | .05 | .01 | .04 | .06 | .00 | .06 | - .01 | .08 | - .02 | .04 | - .02 | .05 | - .03 | - .10* | .09 |

* *p* < .05.

^a^ RAT, random association task

^b^ Number of self-talks participants wrote in the experimental groups or the number of possible learning activity participants wrote in control group.

^c^ Time participants wrote self-talk or possible learning activity.
